# Correlation between time on target and glycated hemoglobin in people with diabetes mellitus: systematic review *

**DOI:** 10.1590/1518-8345.6655.4089

**Published:** 2023-12-04

**Authors:** Rafael Aparecido Dias Lima, Daiane Rubinato Fernandes, Rute Aparecida Casas Garcia, Lucas Ariel da Rocha Carvalho, Renata Cristina de Campos Pereira Silveira, Carla Regina de Souza Teixeira

**Affiliations:** 1 Universidade de São Paulo, Escola de Enfermagem de Ribeirão Preto, Centro Colaborador de la OPS/OMS para el Desarrollo de la Investigación en Enfermería, Ribeirão Preto, SP, Brasil.; 2 Becaria de la Coordenação de Aperfeiçoamento de Pessoal de Nível Superior (CAPES), Brasil.; 3 Universidade Federal de Minas Gerais, UniEscola de Engenharia, Belo Horizonte, MG, Brasil.

**Keywords:** Diabetes Mellitus, Glycated Hemoglobin A, Blood Glucose Self-Monitoring, Continuous Glucose Monitoring, Glycemic Control, Systematic Review, Diabetes Mellitus, Hemoglobina A Glicosilada, Automonitoreo de la Glucemia, Monitorización Continua de la Glucemia, Control Glucémico, Revisión Sistemática, Diabetes Mellitus, Hemoglobina A Glicada, Automonitorização da Glicemia, Monitorização Contínua da Glicemia, Controle Glicêmico, Revisão Sistemática

## Abstract

to analyze the correlation between time on target and glycated hemoglobin in people living with diabetes mellitus and carrying out continuous blood glucose monitoring or self-monitoring of capillary blood glucose.

systematic review of etiology and risk based on JBI guidelines and reported according to Preferred Reporting Items for Systematic Reviews and Meta- Analyses, covering six databases and grey literature. The sample included 16 studies and methodological quality was assessed using JBI tools. Protocol registered in the Open Science Framework, available at https://doi.org/10.17605/OSF.IO/NKMZB.

time on target (70-180 mg/dl) showed a negative correlation with glycated hemoglobin, while time above target (>180 mg/dl) showed a positive correlation. Correlation coefficients ranged between -0.310 and -0.869 for time on target, and between 0.66 and 0.934 for time above target. A study was carried out on a population that performed self-monitoring.

there is a statistically significant correlation between time on target and time above target with glycated hemoglobin. The higher the proportion in the adequate glycemic range, the closer to or less than 7% the glycated hemoglobin will be. More studies are needed to evaluate this metric with data from self-monitoring of blood glucose.

## Abstract (es)

analizar la correlación entre el tiempo en rango y la hemoglobina glicosilada de personas que viven con diabetes mellitus y realizan la monitorización continua de la glucemia o el automonitoreo de la glucemia capilar

revisión sistemática de etiología y riesgo basada en las directrices del JBI e informada según los *Preferred Reporting Items for Systematic Reviews and Meta-Analyses*, abarcando seis bases de datos y la literatura gris. La muestra incluyó 16 estudios y la calidad metodológica fue evaluada utilizando las herramientas del JBI. Protocolo registrado en *Open Science Framework*, disponible en https://doi.org/10.17605/OSF.IO/NKMZB.

tiempo en rango (70-180 mg/dl) mostró una correlación negativa con la hemoglobina glicosilada, mientras que el tiempo por encima del rango (>180 mg/dl) mostró una correlación positiva. Los coeficientes de correlación variaron entre -0,310 y -0,869 para el tiempo en rango, y entre 0,66 y 0,934 para el tiempo por encima del rango. Un estudio se realizó en una población que hacía el automonitoreo.

hay una correlación estadísticamente significativa entre el tiempo en rango y el tiempo por encima del rango con la hemoglobina glicosilada. Cuanto mayor sea la proporción en el rango glucémico adecuado, más cerca o por debajo del 7% estará la hemoglobina glicosilada. Se necesitan más estudios que evalúen esta métrica con datos del automonitoreo de la glucemia.

## Abstract (pt)

analisar a correlação entre o tempo no alvo e a hemoglobina glicada de pessoas que vivem com diabetes mellitus e realizam a monitorização contínua da glicemia ou a automonitorização da glicemia capilar.

revisão sistemática de etiologia e de risco pautada nas diretrizes do JBI e reportada conforme *Preferred Reporting Items for Systematic Reviews and Meta-Analyses*, abrangendo seis bases de dados e a literatura cinzenta. A amostra incluiu 16 estudos e a qualidade metodológica foi avaliada utilizando as ferramentas do JBI. Registrado protocolo no *Open Science Framework*, disponível em https://doi.org/10.17605/OSF.IO/NKMZB.

tempo no alvo (70-180 mg/dl) apresentou correlação negativa com a hemoglobina glicada, enquanto o tempo acima do alvo (>180 mg/dl) mostrou correlação positiva. Os coeficientes de correlação variaram entre -0,310 e -0,869 para o tempo no alvo, e entre 0,66 e 0,934 para o tempo acima do alvo. Um estudo foi efetuado com população que realizava a automonitorização.

há correlação estatisticamente significativa entre o tempo no alvo e o tempo acima do alvo com a hemoglobina glicada. Quanto maior a proporção na faixa glicêmica adequada, mais próxima ou inferior a 7% estará a hemoglobina glicada. São necessários mais estudos que avaliem essa métrica com dados da automonitorização da glicemia.

**Highlights:**

**(1)** All studies showed a significant correlation between time on target and HbA1c.

**(2)** The greater the proportion of time on target, the closer to 7% the HbA1c will be.

**(3)** Possibility to use time on target in blood glucose self-monitoring data.

**(4)** Assessment of patients’ glycemic control in the short, medium and long term.

## Introduction

Blood glucose monitoring is considered a fundamental strategy for preventing complications from diabetes mellitus (DM), resulting in an improvement in the quality of life of people living with this chronic disease ^(^ 1 ^)^. Currently, with the advent of new technologies, continuous blood glucose monitoring (CBGM) is emphasized using sensors applied subcutaneously, which allow uninterrupted measurement of current and real blood glucose levels ^(^ 2 ^)^. 

Systematic reviews were developed with a view to comparing the effectiveness of CBGM and self-monitoring of capillary blood glucose (SMCBG) in the management of glycemic control in DM. These reviews highlight that CBGM offers significant advantages in relation to SMCBG, such as a greater amount of data, continuous assessment of glycemia and detection of glycemic patterns imperceptible by SMCBG ^(^ 3 ^-^ 5 ^)^, highlighting the emergence of new metrics for the assessment of glycemic control, such as time on target ^(^ 2 ^)^. 

Time on target refers to the time spent in an individual’s given glycemic range, generally between 70-180 mg/dl, but ideally between 70-140 mg/ dl ^(^ 6 ^)^. Its measurements add important information to analyze the level of glycemic control, in addition to what is known from glycated hemoglobin (HbA1c), as it is possible to acquire and evaluate data not only regarding hyperglycemia, but also hypoglycemia, effective, therefore, for avoid both micro and macrovascular complications ^(^ 6 ^-^ 7 ^)^. 

Therefore, although HbA1c is widely used as a gold standard indicator to assess glycemic control over time, it does not provide detailed information about daily blood glucose levels ^(^ 8 ^)^. On the other hand, time on target offers a more accurate and individualized perspective on glycemic regulation ^(^ 89 ^)^. Recent studies have suggested that time on target may be a better predictor of clinical outcomes and risk of diabetes complications, compared to HbA1c alone, even suggesting the replacement of this indicator with this new measure ^(^ 10 ^-^ 13 ^)^. 

However, as it is a metric derived from a new technology, its access is still restricted to a small portion of the population with diabetes, mainly those residing in high-income countries ^(^ 14 ^)^. 

In this way, the social inequity of diabetes stands out ^(^ 15 ^)^, since the majority of people living with DM live in low- and middle-income countries ^(^ 15 ^)^ and have financial obstacles in accessing new technologies in managing diabetes. Glycemic control, also widely using self-monitoring of capillary blood glucose (SMCBG), which, despite having limitations in relation to CBGM devices, is ratified in the literature as a fundamental tool in glycemic control through the provision of feedback on the glycemia levels, which facilitates understanding of the impact of specific food choices and physical activities in relation to each patient’s glycemia ^(^ 16 ^)^. 

In this context, it is necessary to identify scientific evidence on the correlation between time on target and HbA1c in people living with type 1 DM (DM1), type 2 DM (DM2) or gestational DM and who undergo CBGM or SMCBG so that we can better understand the relationship between these two metrics in the management of DM and verifying the possibility of the applicability of time on target in SMCBG data, justifying the development of this review, since to date no reviews with this purpose have been found in the literature.

From this perspective, the objective of this review was to analyze the correlation between time on target and HbA1c in people living with DM and who perform CBGM or SMCBG.

## Method

### Type of study

A systematic review is a research method that supports evidence-based healthcare. In this sense, this review was carried out according to the JBI approach, aiming to synthesize evidence on the correlation between time on target and HbA1c in people with DM. Association questions commonly address etiological or prognostic problems. Although there is no universally recognized methodology for systematic reviews on etiology and risk, these reviews provide valuable information for healthcare professionals and decision makers and can influence health outcomes. The systematic review of etiological studies is essential in the context of public health, as it guides health care planning, resource allocation and disease prevention strategies ^(^ 17 ^-^ 18 ^)^. The method was conducted in a rigorous and transparent way to identify, select and critically appraise the included primary studies. 

Therefore, this review followed a sequence of steps: formulation of the research question; definition of inclusion and exclusion criteria; search and selection of studies; assessment of methodological quality; data extraction, analysis and synthesis of studies; and presentation and interpretation of results ^(^ 18 ^)^. It was reported according to the items proposed by Preferred Reporting Items for Systematic Reviews and Meta-Analysis (PRISMA) ^(^ 19 ^)^. 

The protocol for this systematic review was previously published on the Open Science Framework platform, whose registration is available at https://doi.org/10.17605/OSF.IO/NKMZB

### Eligibility criteria

To define the eligibility criteria, the PEO (Population, Exposure and Outcome) ^(^ 17 ^-^ 18 ^)^, together with the formulation of the research question. In this systematic review, the acronym PEO was used as follows: P (Population) refers to people with type 1, type 2 or gestational DM; E (Exposure) involves CBGM or SMCBG; (Outcome) covers the correlation between HbA1c and time on target. 

The research question outlined was: “what is the correlation between time on target and HbA1c in people living with type 1, type 2 or gestational DM who underwent SMCBG or CBGM?”

The inclusion criteria for selecting the studies were: people diagnosed with type 1, type 2 or gestational DM who used SMCBG or CBGM as a strategy for glycemic control, in addition to having a laboratory-collected HbA1c sample, correlated with time on target. The studies considered in the research were those published in English, Portuguese and Spanish, in any publication period and obtained in full.

On the other hand, the exclusion criteria were applied to studies that involved people with unspecified DM, that correlated glycated albumin with time on target, used estimated HbA1c instead of laboratory collected, or consisted of case reports, case series, secondary studies (other reviews), editorials, letters to the editor, books, book chapters, guidelines, expert opinion articles, experience reports, conference proceedings and abstracts, dissertations and theses.

### Data source

The studies were tracked using the following electronic databases: Cumulative Index to Nursing and Allied Health (CINAHL), Cochrane Library, Excerpta Doctor Data base (Embase) , Latin American and Caribbean Literature in Health Sciences (LILACS), PubMed and Scopus . Additionally, grey literature was explored through Google Scholar .

To build the search strategy, controlled descriptors and their synonyms were used: “diabetes mellitus”, “blood glucose self- monitoring”, “continuous glucose monitoring”, “time in range”, “glycated hemoglobin A”, associated with Boolean operators AND or OR, grouped and adapted according to the specificities of each database in this review.

The search strategy was technically evaluated by a librarian, and once completed, tests were carried out to check whether there was sensitivity to the research question to be answered. The detailed tests and terms of the constructed search strategy are presented in Figure 1. 

**Figure 1 -:** Search strategy according to electronic databases. Ribeirão Preto, SP, Brazil, 2022

Database	Search strategy
PubMed	((((“Diabetes Mellitus”[MeSH Terms]) OR “Diabetes Mellitus” [All Fields] OR “Diabetes Mellitus, Type 1”[MeSH Terms] OR “Diabetes Mellitus, Type 1”[All Fields] OR “Diabetes Mellitus, Type 2”[MeSH Terms] OR “Diabetes Mellitus, Type 2”[All Fields] OR “Diabetes, Gestational”[MeSH Terms] OR “Diabetes, Gestacional”[All Fields]))) AND ((“Blood Glucose Self-Monitoring”[MeSH Terms] OR “Blood Glucose Self-Monitoring”[All Fields] OR “Home Blood Glucose Monitoring”[All Fields] OR “Continuous Glucose Monitoring”[All Fields])) AND ((“Time in range”[All Fields] OR “Time Above Range”[All Fields] OR “Time Below Range”[All Fields])) AND ((“Glycated Hemoglobin A”[MeSH Terms] OR “Glycated Hemoglobin A”[All Fields] OR “Hb A1c”[All Fields] OR “Glycated Hemoglobin”[All Fields]))
Scopus	(( ‘diabetes AND mellitus’ OR ‘diabetes AND mellitus, AND type AND 1’ OR ‘diabetes AND mellitus, AND type AND 2’ OR ‘diabetes, AND gestational’ ) AND ( ‘blood AND glucose AND self-monitoring’ OR ‘home AND blood AND glucose AND monitoring’ OR ‘continuous AND glucose AND monitoring’ ) AND ( ‘time AND in AND range’ OR ‘time AND above AND range’ OR ‘time AND below AND range’ ) AND ( ‘glycated AND hemoglobin AND a’ OR ‘hb AND a1c’ OR ‘glycated AND hemoglobin’ ))
Embase	(‘diabetes mellitus’ OR ‘diabetes mellitus, type 1’ OR ‘diabetes mellitus, type 2’ OR ‘diabetes, gestational’) AND (‘blood glucose self-monitoring’ OR ‘home blood glucose monitoring’ OR ‘continuous glucose monitoring’) AND (‘time in range’ OR ‘time above range’ OR ‘time below range’) AND (‘glycated hemoglobin a’ OR ‘hb a1c’ OR ‘glycated hemoglobin’)
CINAHL	“Diabetes Mellitus” OR “Diabetes Mellitus, Type 1” OR “Diabetes Mellitus, Type 2” OR “Diabetes, Gestational” AND “Blood Glucose Self-Monitoring” OR “Home Blood Glucose Monitoring” OR “Continuous Glucose Monitoring” AND “Time in range” OR “Time Above Range” OR “Time Below Range” AND “Glycated Hemoglobin A” OR “Hb A1c” OR “Glycated Hemoglobin”
Cochrane Library	0 Trials matching “Diabetes Mellitus” OR “Diabetes Mellitus, Type 1” OR “Diabetes Mellitus, Type 2” OR “Diabetes, Gestational” AND “Blood Glucose Self-Monitoring” OR “Home Blood Glucose Monitoring” OR “Continuous Glucose Monitoring” AND “Time in range” OR “Time Above Range” OR “Time Below Range” AND “Glycated Hemoglobin A” OR “Hb A1c” OR “Glycated Hemoglobin” in Title Abstract Keyword
LILACS	“Diabetes Mellitus” OR “Diabetes Mellitus, Type 1” OR “Diabetes Mellitus, Type 2” OR “Diabetes, Gestational” OR “Diabetes Mellitus, Tipo 1” OR “Diabetes Mellitus, Tipo 2” OR “Diabetes, Gestacional” AND “Blood Glucose Self-Monitoring” OR “Automonitorização da Glicemia Capilar” OR “Automonitorizacion de la Glucosa” OR “Continuous Glucose Monitoring” OR “Monitorização Continua da Glicose” OR “Monitorizacion Continua de Glucosa” AND “Time in range” OR “Tempo no intervalo” OR “Tiempo em Rango” AND “Glycated Hemoglobin A” OR “Hemoglobina A Glicada” OR “Hemoglobina A Glucada”
Google Scholar	(Diabetes Mellitus) OR (Diabetes Mellitus Type 1) OR (Diabetes Mellitus, Type 2) OR (Diabetes Gestational) AND (Blood Glucose Self-Monitoring) OR (Home Blood Glucose Monitoring) OR (Continuous Glucose Monitoring) AND (Time in range) OR (Time Above Range) OR (Time Below Range) AND (Glycated Hemoglobin A) OR (Hb A1c) OR (Glycated Hemoglobin)

The search results were exported to the EndNote Basic reference manager ^(^ 20 ^)^ online version to remove duplicate references and then imported into the Rayyan platform, which can be accessed via the website https://rayyan.qcri.org ^(^ 21 ^)^. 

Rayyan platform ^(^ 21 ^)^, the studies were first evaluated by reading the title and abstract, by two reviewers independently and blinded, according to the eligibility criteria. The studies considered eligible were then analyzed by reading the full text. In case of disagreement between the reviewers, a third reviewer with expertise on the topic was consulted. 

### Period

The search in electronic databases was carried out on September 20, 2021 and updated on June 20, 2023.

### Process used to extract and analyze information from selected studies

Data from the studies were collected using a pre-established standard form, once again independently by two researchers, which includes: reference, year of publication and country of study, journal and its impact factor, objective, study design, sample size, main results and, therefore, the studies were analyzed qualitatively, synthesizing the evidence in a descriptive way.

It is noteworthy that the synthesis of evidence occurred through correlation values between HbA1c and time on target, as well as the proportions at a given time on target and the corresponding HbA1c.

After completing this process, the two researchers compared the data obtained and resolved any disagreements through discussion and consensus. In situations where there was disagreement, a third researcher specialized in the topic in question was consulted to obtain a final decision.

### Assessment of methodological quality

The methodological quality assessment was carried out using the tools provided by JBI ^(^ 18 ^)^. These tools incorporate a critical process of evaluating research evidence, their main objective being to evaluate the methodological quality of a study and determine the extent to which this study presented the possibility of bias in its design, conduct and analysis ^(^ 18 ^)^. 

Before the critical evaluation of the studies began, decisions about the responses were discussed among the reviewers. Thus, the greater the number of “yes” responses to the items evaluated in the tool, the greater the methodological quality of the study. This step was also carried out independently and blinded by two reviewers. The third reviewer was called to resolve possible conflicts in this assessment ^(^ 22 ^)^. 

### Ethical aspects

As it is a secondary study, submission to the Research Ethics Committee (REC) is not mandatory. There are no conflicts of interest that could compromise the analysis of the results of this work.

## Results

At the end of the searches carried out in the electronic databases, 377 records were identified, of which 72 were removed because they were duplicates. Subsequently, 305 documents were analyzed by reading the title and abstract. A total of 27 studies were selected for full-text reading.

**Figure 2 -:**
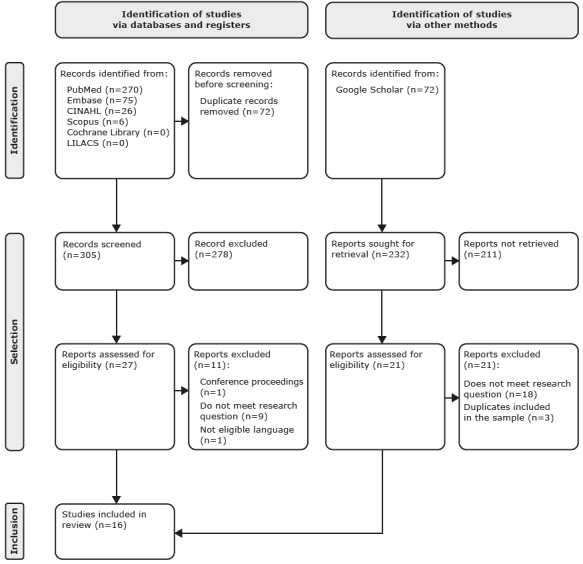
Flowchart of the systematic review, according to PRISMA (2020)

After reading in full, 11 articles were excluded following the selection criteria. At the end of the selection process, 16 studies were selected to compose the systematic review and subjected to descriptive analysis, as described in Figure 2. 

Regarding grey literature, of the 232 studies selected, 211 were excluded after reading the title and abstract. Therefore, 21 records were read in full, none of which were selected to compose this systematic review because they did not answer the question or because they were duplicate articles already selected in scientific databases, as shown in Figure 2. 

The characteristics of the studies included in this systematic review are described in detail in Figure 3. 

**Figure 3 -:** Summary of studies included in the systematic review. Ribeirão Preto, SP, Brazil, 2022

Author, year, journal (impact factor), country, study design and sample	Objective	Intervention	Main results
Beck, et al. (2019) ^(^ 22 ^)^ Journal of Diabetes Science and Technology (1,306) USA Cross-sectional study 545 people ≥ 18 years old living with DM1*	To evaluate aspects of glycemia metrics by CBGM ^†^ and their relationship with HbA1c ^‡^ aiming to facilitate the effective use of CBGM ^†^ optimizing DM management ^§^.	Participants underwent 6 months of CBGM ^†^. The main CBGM ^†^ metrics included in the analyzes were: time on target (70-180 mg/dl); optimal time on target (70-140 mg/dl); time above target (>180 mg/dl); time above target (>250 mg/dl), time below target (<70 mg/dl) and time below target (<54 mg/dl). Spearman partial correlation coefficient.	Moderate correlation between time on target (70-180 mg/dl) and HbA1c ^‡^ (-0.73 at month 6). Time on target (70-180 mg/dl) of 50% on average was associated with an HbA1c ^‡^ level of about 8%. Time on target (70-180 mg/dl) of 30% on average was associated with an HbA1c ^‡^ of about 8.7%. Time on target (70-180 mg/dl) of 70% on average was associated with an HbA1c ^‡^ of about 7%.
Hirsch, et al. (2019) ^(^ 23 ^)^ Diabetic Medicine (4,359) USA Cross-sectional study 530 people ≥ 18 years old (455 with DM1* and 75 with DM2 ^||^ on insulin treatment)	To identify clinically useful associations between HbA1c ^‡^ levels and CBGM ^†^-derived metrics.	≥ 2 weeks of blood glucose data from CBGM ^†^ sensors were analyzed. The main metrics included in the analyzes were: time on target (70-180 mg/dl), time above target (>250 mg/dl) and time below target (<70 mg/dl), correlating these variables with HbA1c ^‡^ (corresponding to the last 3 months of study participation). Multiple regression analysis.	Strong inverse correlation between HbA1c ^‡^ and time on target (70-180 mg/dl) (-0.75), each 10% change in time on target was associated with a 0.7% change in HbA1c ^‡^. Strong positive correlations between HbA1c ^‡^ and time above target level 2 (>250 mg/dl) (0.72). Weak inverse correlation between HbA1c ^‡^ and time below target (<70 mg/dl) (-0.39). Of 139 subjects with time on target ≥70%, 111 had HbA1c ^‡^ ≤7%.
Peterson, et al. (2019) ^(^ 24 ^)^ Pediatric Diabetes (4,866) Sweden Cross-sectional study 105 children and adolescents ≤18 years old with DM1*	To analyze the relationship between time on target and HbA1c ^‡^.	Data on CBGM ^†^ blood glucose values from the last 30, 60 and 90 days were analyzed. The metrics included in the analyzes were : time on target (70-180 mg/dl); optimal time on target (70-140 mg/dl); time above target (>180 mg/dl) and time below target (<70 mg/dl). Regression analysis was used to estimate the association between optimal time on target, time on target, and HbA1c ^‡^. Both linear and quadratic models were calculated and the explained variance (R2 *)* between the two models was compared	Significant linear relationship between optimal time on target and HbA1c ^‡^ ( *R* ^2^ *=* 0.63, p < 0.0001). Significant linear (B = 0.51, *R* ^2^ *=* 0.68, p < 0.0001) and non-linear ( *R* ^2^ *=* 0.70, p < 0.001) relationship between time above target (>180 mg/dl) in the last 60 days and HbA1c ^‡^. Weak linear relationship between time below target (<70 mg/dl) and HbA1c ^‡^ in the last 30 days ( *R* ^2^ *=* 0.08), 60 days ( *R* ^2^ *=* 0.06), and 90 days ( *R* ^2^ *=* 0,01). HbA1c ^‡^ of 6.5% equals 50.0% optimal time on target (70-140 mg/dl)
Tsuchiya, et al. (2020) ^(^ 25 ^)^ Endocrine Journal (2,349) Japan Cross-sectional study 156 people ≥ 18 years old with DM2 ^||^ being treated with insulin or oral hypoglycemic medication	To characterize the relationship between Daily Glycemic Variability assessed by CBGM ^†^ and Visit-to-Visit Glycemic Variability in Japanese patients with DM2 ^||^.	5-day CBGM ^†^ values. HbA1c ^‡^ was obtained from patient records. Time on target (70-180 mg/dl), time above target (> 180 mg/dl) and time below target (<70 mg/dl) were evaluated. Spearman correlation coefficient and multiple regression analysis.	Time on target (70-180 mg/dl) was significantly correlated with HbA1c ^‡^ (–0.310, p < 0.01). The association between time on target (70-180 mg/dl) and HbA1c ^‡^ remained significant after adjusting for age, sex and BMI in multiple regression analysis (β = –0.300, p < 0.001).
Cutruzzola, et al. (2020) ^(^ 26 ^)^ Nutrition, Metabolism, and Cardiovascular Diseases (4,222) Italy Cross-sectional study 233 people ≥18 years old with DM ^§^ treated with insulin (197 with DM1* and 36 with DM2 ^||^ on insulin treatment)	To evaluate the association between HbA1c ^‡^ and percentage of points spent in time on target calculated from SMCBG values in patients with DM1* and DM2 ^||^ treated with insulin.	SMCBG data was downloaded and two distinct periods were selected to calculate new metrics: 2 months and 2 weeks before the last HbA1c ^‡^ available. The metrics used were point on target (70-180 mg/dl), point above target (>180 mg/dl) and point below target (<70 mg/dl). Univariate linear regression analysis.	Significant and negative correlation (R= -0.72) between HbA1c ^‡^ and percentage point on target measured over 2 weeks and 2 months in individuals with DM1* and DM2 ^||^). A significant and inverse correlation (R= -0.18) was found between HbA1c ^‡^ and the point below the target, and a significant and positive correlation (R= 0.75) between HbA1c ^‡^ and the point above the target. Point on target equal to 70% corresponded to an HbA1c ^‡^ value of approximately 7%. Each change in target point by 10% corresponded to a change in HbA1c ^‡^ of 0.4%.
Urakami, et al. (2020) ^(^ 27 ^)^ Endocrine Journal (2,860) Japan Cross-sectional study 85 children and adolescents ≤18 years old with DM1*	To assess the significance of international consensus recommendations on metrics derived from the CBGM ^†^ in Japanese children and adolescents with DM1*.	28-day CBGM ^†^ blood glucose data. The relationship between CBGM-derived metrics ^†^ was evaluated: time on target (70-180 mg/dl), time above target (>180 mg/dl) and time below target (<70 mg/dl) with the HbA1c ^‡^. Statistical analysis: Pearson correlation coefficients.	HbA1c ^‡^ levels showed a highly inverse correlation with time on target (70-180 mg/dl) (-0.869, p < 0.0001), highly positive correlation with time above target (>180 mg/dl) (0.934, p < 0.0001) and weakly inverse correlation with time below target (<70 mg/dl) (–0.351, p = 0.001). HbA1c ^‡^ of 7.0% corresponded to a time on target of 55.1%.
Valenzano, et al. (2021) ^(^ 28 ^)^ BMJ Open Diabetes (3,388) Italy Cross-sectional study 59 people between 20 and 60 years old with DM1*	To contribute, with data from around the world, to an understanding of the relationship between Time on target and HbA1c ^‡^.	Participants used CBGM ^†^ devices for 1 year. Follow-up visits were performed after 90, 180, and 365 days from baseline and percentage time on target (70-180 mg/dl) assessed for the 90-day period prior to each visit. Pearson correlation coefficient and univariate linear regression.	Strong correlation (-0.73) between HbA1c ^‡^ and time on target (70-180 mg/dl) based on 90-day CBGM ^†^ data under real-life conditions. There was a 0.5% decrease in HbA1c ^‡^, from 7.5% to 7.0% in an improvement in percentage time on target (70-180 mg/dl) from 52.9% to 58.8%.
Kuroda, et al. (2021) ^(^ 29 ^)^ Journal of Diabetes Investigation (4,232) Japan Cohort study 281 people between 40 and 75 years old with DM2 ^||^ being treated with insulin or oral hypoglycemic drugs	To investigate the relationship between Time on target, glycemic variability and characteristics of patients with DM2 ^||^	Blood glucose sensor data was primarily collected over a 10-day period (≥70% of 14-day CBGM data). Time on target (70-180 mg/dl) and time below target (<70 mg/dl) were used as objective variable, and multiple regression analysis was performed using variables including age, sex, disease duration and HbA1c ^‡^, as explanatory variables.	The results showed that HbA1c ^‡^ (standard partial regression coefficient; β = -0.573, p < 0.001), disease duration ( β = -0.160, p = 0.003) were useful explanatory factors for time on target (70-180 mg/ dl). HbA1c ^‡^ (β = −0.431, p < 0.001) and use of medications with a high risk of hypoglycemia (β = 0.147, p = 0.030) were useful explanatory factors for time below target.
Ling, et al. (2021) ^(^ 30 ^)^ The Journal of Clinical Endocrinology and Metabolism (5,958) China Cross-sectional study 98 pregnant women > 18 years old with DM1*	To investigate the relationship between MCG ^†^ and laboratory HbA1c ^‡^ metrics in pregnant women with DM1*.	CBGM ^†^ data during pregnancy and postpartum. Data was collected to calculate Time on Target (60-140 mg/dl), Time on Target (70-180 mg/dl), Time Above Target (>140 mg/dl), Time Above Target (>180 mg/dl), time below target (<60 mg/dl) and time below target (<54 mg/dl). Spearman coefficient analysis.	The analysis showed a negative correlation between time on target (70-180 mg/dl) and HbA1c ^‡^ during pregnancy (-0.429) and in the postpartum period (-0.766). HbA1c ^‡^ of 6.0%, 6.5% and 7.0% were equivalent to a time on target of 78%, 74% and 69%, respectively.
Den Braber, et al. (2021) ^(^ 31 ^)^ Diabetes Care (19,112) Netherlands Cohort study 79 people ≥18 years old with DM2 ^||^ treated with insulin	To investigate blood glucose variations associated with HbA1c ^‡^ in patients with DM2 ^||^ treated with insulin.	CBGM ^†^-derived parameters for 2 weeks. The following metrics were analyzed: time on target (70-180 mg/dl); time above target (>180 mg/dl); time above target (>250 mg/dl), time below target (<70 mg/dl) and time below target (<54 mg/dl). Best-fit regression analyzes with 95% prediction intervals.	Correlation between HbA1c ^‡^ and time on target (70-180 mg/dl) R ^2^ = 0.65. Time below target (<70 mg/dl) decreased progressively in increasing HbA1c categories ^‡^ while time above target (>180 mg/dl) increased progressively.
Bosoni, et al. (2021) ^(^ 32 ^)^ Journal of Pediatric Endocrinology and Metabolism (1,520) Italy Cross-sectional study 23 children and adolescents ≤18 years old with DM1*	Investigate the relationship between CBGM ^†^-derived glycemic metrics and HbA1c ^‡^.	CBGM ^†^ blood glucose data for 30, 60 and 90 days. The following metrics from the CBGM ^†^ were considered: Time on target (70-180 mg/dl); optimal time on target (70-140 mg/dl); time above target (>180 mg/dl); time above target (>250 mg/dl), time below target (<70 mg/dl) and time below target (<54 mg/dl). Linear regression analysis.	Time on target (70-180 mg/dl) and optimal time on target (70-140 mg/dl) had a negative linear relationship with HbA1c ^‡^ (R ^2=^ 0.88). Time above target (>180 mg/dl) and time above target (>250 mg/dl) showed a positive linear relationship with HbA1c ^‡^ (R ^2=^ 0.75). For an HbA1c ^‡^ ≤ 7%, a Time on Target (70-180 mg/dl) of 65% and an Optimal Time on Target (70-140 mg/dl) of 48% are required.
Babaya, et al. ( 2021) ^(^ 33 ^)^ Scientific Reports (4,996) Japan Cross-sectional study 19 people ≥18 years old with DM1*	Clarify the relationship between various CBGM ^†^ metrics and HbA1c ^‡^.	Data obtained by CBGM ^†^ during 4 months. CBGM ^†^ data from 120, 90, 60, 30 and 7 days were used to calculate time on target (70-180 mg/dl); optimal time on target (70-140 mg/dl); time above target (>180 mg/dl); time above target (>250 mg/dl), time below target (<70 mg/dl) and time below target (<54 mg/dl). Univariate regression analysis.	Time on target (70-180 mg/dl) was strongly correlated with HbA1c ^‡^ values (R²= 0.888; p <0.0001). There was a strong positive correlation between time above target (>180 mg/dl) and HbA1c ^‡^ (R²= 0.944; p<0.0001). HbA1c ^‡^ of approximately 7% corresponded to a time on target (70-180 mg/dl) of 74%.
Ohigashi, et al. (2021) ^(^ 34 ^)^ *Journal of Diabetes* *Investigation* (3,681) Japan Cohort study 167 people between 20 and 80 years old with DM ^§^, 67 with DM1* and 100 with DM2 ^||^ using oral hypoglycemic medication and/or insulin	Investigate relationships between CBGM ^†^ and HbA1c ^‡^ metrics.	14-day CBGM ^†^ data. HbA1c ^‡^ was collected in the laboratory on the day of CBGM ^†^ application. Time on target (70-180 mg/dl) was calculated; time above target (>180 mg/dl); time above target (>250 mg/dl), time below target (<70 mg/dl) and time below target (<54 mg/dl). Spearman correlation coefficient and multiple regression.	In patients with DM1* and DM2||, HbA1c ^‡^ was negatively (-0.62) correlated with time on target (70-180 mg/dl). Strong positive correlation between time above target (>180 mg/dl) and HbA1c ^‡^ (0.66). In patients with DM1*, a time on target of 70% corresponded to an HbA1c ^‡^ of 6.9%. In patients with DM2 ^||^, a time on target of 70% corresponded to an HbA1c ^‡^ of 7.1%.
Díaz-Soto, et al. (2021) ^(^ 35 ^)^ Endocrinology , Diabetes and Nutrition (1,833) Spain Cross-sectional study 195 people with DM1* (70 <20 years and 125 >20 years)	To evaluate the relationship between HbA1c ^‡^, Time on Target and glycemic variability in a cohort of pediatric and adult patients with DM1* and CBGM ^†^.	CBGM ^†^ 90-day blood glucose data. Time on target (70-180 mg/dl) was calculated; time above target (>180 mg/dl) and time below target (<70 mg/dl). The different times were correlated with HbA1c ^‡^. Statistical analysis: Pearson’s linear correlation coefficient and multiple regression.	There was a strong negative linear correlation (−0.746; p<0.001) between time on target and HbA1c ^‡^.
Kurozumi, et al. (2022) ^(^ 36 ^)^ Diabetes Research and Clinical Practice (8,180) Japan Cross-sectional study 999 people with DM2 ^||^ aged ≥ 30 and < 80 years treated with insulin or oral hypoglycemic agent	Define the relationship between Time on Target and HbA1c levels ^‡^ in patients with DM2 ^||^.	14-day CBGM ^†^ data. Correlation was performed between time on target (70-180 mg/dl); time above target (>180 mg/dl); time above target (>250 mg/dl), time below target (<70 mg/dl) and time below target (<54 mg/dl) with the last laboratory- collected HbA1c ^‡^. Statistical analysis: Pearson correlation coefficient and linear regression.	HbA1c ^‡^ significantly correlated with time on target (70-180 mg/dl) (-0.71). An HbA1c ^‡^ of 7% was associated with a time on target (70-180 mg/dl) of 80.64%. HbA1c ^‡^ decreased by 0.3% for every 10% increase in time on target.
Alarcon, et al. (2022) ^(^ 37 ^)^ Endocrinology, Diabetes and Nutrition (1,833) Spain Cross-sectional study 252 people with DM1* aged between 15-79 years	Evaluate the correlation between MCG ^†^ and HbA1c ^‡^ parameters	14-day CBGM ^†^ blood glucose data Correlated with time on target (70-180 mg/dl); time above target (>180 mg/dl); and time below target (<70 mg/dl) with the last laboratory-collected HbA1c ^‡^. Statistical analysis: Correlation with the Pearson test and linear regression.	Statistically significant correlation between time on target (70-180 mg/dl) and HbA1c ^‡^ (-0.623; p < 0.01).

*Type 1 Diabetes Mellitus; ^†^Continuous Blood Glucose Monitoring; ‡Glycated Hemoglobin A; §Diabetes Mellitus; ^||^Type 2 Diabetes Mellitus

The majority of studies were carried out in developed countries, with 43.7% of studies coming from European countries, including Italy (n=3), Spain (n=2), the Netherlands (n=1) and Sweden (n= 1). Likewise, 43.7% of studies come from Asian countries, including Japan (n=6) and China (n=1). Finally, 12.6% of the studies come from North America, specifically the United States (n=2).

This systematic review shows that the topic in question has a constantly growing scientific base, with the first articles published in 2019 and the most recent in 2022.

Articles that met the inclusion criteria were subjected to a critical assessment of their methodological quality, according to the tools appropriate to the study design adopted. The majority of studies (75%, n=13) adopted an analytical cross-sectional research design. It is important to note that only one of the studies evaluated presented information related to the identification of confounding factors and none of these studies addressed possible strategies for coping with these factors, as shown in Figure 4. 

**Figure 4 -:** Methodological quality assessment according to the JBI Critical tool Appraisal Tool according to the type of study (cross-sectional studies). Ribeirão Preto, SP, Brazil, 2022

Studies	Q1*	Q2 ^†^	Q3 ^‡^	Q4 ^§^	Q5 ^||^	Q6 ^¶^	Q7**	Q8 ^††^
Beck, et al. (2019)	S	S	S	S	UN	UN	S	S
Hirsch, et al. (2019)	S	S	UN	S	N	N	S	UN
Peterson, et al. (2019)	S	S	S	S	UN	UN	S	S
Tsuchiya, et al. (2020)	S	S	N	S	S	N	S	S
Cutruzzola, et al. (2020)	S	S	S	S	N	N	S	S
Urakami, et al. (2020)	S	S	N	S	UN	UN	S	S
Valenzano, et al. (2021)	S	S	S	S	UN	UN	S	S
Ling, et al. (2021)	S	S	UN	S	UN	UN	S	S
Bosoni, et al. (2021)	N	S	S	S	N	N	S	S
Babaya, et al. (2021)	S	N	S	S	N	N	S	S
Díaz-Soto, et al. (2022)	S	S	S	S	UN	UN	S	S
Kurozumi, et al. (2022)	S	S	S	S	UN	UN	S	S
Alarcon, et al. (2022)	S	S	S	N	N	N	S	S
%	**92**	**92**	**61**	**92**	**8**	**0**	**100**	**92**

*Q1: Were the sample inclusion criteria clearly defined?; ^†^Q2: Were the study subjects and environment described in detail?; ^‡^Q3: Was exposure measured validly and reliably?; §Q4: Were objective and standardized criteria used to measure the condition?; ^||^Q5: Have confounding factors been identified?; ¶Q6: Have strategies been established to deal with confounding factors?; **Q7: Were the results measured validly and reliably?; ^††^Q8: Was appropriate statistical analysis used?; S: Yes; N: No; UN: Uncertain

On the other hand, the remaining studies (n=3) followed a cohort design. It is important to highlight that none of these studies addressed the issues of identifying and resolving potential confounding factors or provided strategies for dealing with cases of incomplete follow-up, as detailed in Figure 5. 

**Figure 5 -:** Methodological quality according to the JBI Critical Appraisal Tool according to the type of study (cohort studies). Ribeirão Preto, SP, Brazil, 2022

Studies	Q1*	Q2 ^†^	Q3 ^‡^	Q4 ^§^	Q5 ^||^	Q6 ^¶^	Q7**	Q8 ^††^	Q9 ^‡‡^	Q10 ^§§^	Q11 ^||||^
Kuroda, et al. (2021)	UN	UN	S	N	N	S	S	S	S	N	S
Ohigashi, et al. (2021)	UN	UN	S	N	N	S	S	S	UN	N	S
Den Braber, et al. (2021)	S	S	S	UN	UN	S	S	S	UN	N	S
%	**33**	**33**	**100**	**0**	**0**	**100**	**100**	**100**	**33**	**0**	**100**

*Q1: Were the two groups similar and recruited from the same population?; †Q2: Were exposures measured similarly to assign people to exposed and unexposed groups?; ‡Q3: Was exposure measured validly and reliably?; §Q4: Were confounding factors identified?; ^||^Q5: Have strategies for dealing with confounding factors been stated?; ¶Q6: Were the groups/participants free from the outcome at the beginning of the study (or at the time of exposure)?; **Q7: Were the results measured validly and reliably?; ^††^Q8: Was the follow-up time reported and sufficient for the results to occur?; ^‡‡^Q9: Was follow-up complete and, if not, were reasons for loss to follow-up described and explored?; Q10§§: Were strategies used to address incomplete monitoring?; ^||||^Q11: Was appropriate statistical analysis used?; S: Yes; N: No; UN: Uncertain

Regarding the characterization of the population of the studies included in this review, the majority (68.8%; n=11) were adults over 18 years of age. In 18.7% of the studies (n=3), the research was carried out with children and/or adolescents aged up to 18 years, while in 12.5% of the studies (n=2), the participating population was mixed, including children and/or teenagers, as well as adults.

Regarding the collection of glycemic data, the vast majority of studies (93.8%; n=15) used CBGM sensors to obtain glycemia values. Only one study (6.2%) used data from SMCBG.

Regarding the type of diabetes, 56.4% of study participants (n=9) had DM1. In 18.7% of the studies (n=3), participants had DM2 and were using insulin or oral hypoglycemic drugs. In 12.5% of the studies (n=2), the research involved people with DM1 or DM2 using insulin. One study (6.2%) included participants with DM2 using insulin, and another study (6.2%) involved participants with DM1 or DM2 using insulin or oral hypoglycemic agents. It is important to highlight that none of the studies were conducted in a population with gestational diabetes.

Regarding the sample size in each study, variability was observed, with the number of participants varying from 19 to 999 in each study included in this review.

The studies in this review varied in terms of the periods of analysis of blood glucose data. One study used 5 days of data, followed by another with 7 days and a third with 28 days. Six studies adopted a 14-day analysis period. Three studies evaluated 30-day data, while three others used 60-day data. Additionally, five studies analyzed 90-day data, two studies had a 120-day period, and one study used 180-day data.

All 16 included studies addressed the correlation between time on target (70-180 mg/dl) and HbA1c. Three studies (18.7%) also investigated time at optimal target (70-140 mg/dl). 12 studies (75.0%) examined time below target (<70 mg/dl), while 7 studies (43.7%) investigated time below target (<54 mg/dl). Regarding time above target (>180 mg/dl), 14 studies (87.5%) analyzed the correlation with HbA1c, and 7 studies (43.7%) examined time above target (>250 mg/dl). Only one study (6.3%) investigated time on target of 60-140 mg/dl, time below target (<60 mg/dl), and time above target (>140 mg/dl).

Spearman coefficient in conjunction or not with a regression model. In the remaining 43.7% (n=7) of the studies, only regression models, both multiple and univariate, were applied.

All studies showed a correlation between time on target (70-180 mg/dl) and HbA1c: those that used the Spearman coefficient showed a correlation between -0.310 to -0.766; studies that used Pearson’s coefficient showed a correlation between -0.623 and -0.869.

Among the studies that used regression models, we found the following results: one study revealed a significant linear relationship between time on target and HbA1c (R²= 0.63); another study showed a significant negative correlation (R= -0.72); a third study showed a relationship; negative linear with HbA1c (R² >0.88); research found a strong correlation between these two metrics and HbA1c values (R²= 0.888); another study found a correlation of R²= 0.65 between HbA1c and time on target (70-180 mg/dl); Finally, one study concluded that HbA1c ( β = -0.573, p <0.001) was a significant factor correlated with time on target (70-180 mg/dl).

There was also a significant correlation between time above target (>180 mg/dl) and HbA1c with correlation coefficients between 0.66 and 0.934.

## Discussion

In the present systematic review, it was possible to highlight that all the studies analyzed showed a correlation between time on target (70-180 mg/dl) and HbA1c. Using Spearman ^(^ 22 ^,^ 25 ^,^ 30 ^,^ 34 ^)^ and Pearson ^(^ 27 ^-^ 28 ^,^ 35 ^-^ 37 ^)^ coefficients, the correlation ranged from -0.310 to -0.766 and from -0.623 to -0.869, respectively. Regression models also indicated a significant linear relationship between time on target and HbA1c ^(^ 23 ^-^ 24 ^,^ 26 ^,^ 29 ^,^ 31 ^-^ 33 ^)^. Furthermore, there was a significant correlation between time above target (>180 mg/dl) and HbA1c, with correlation coefficients between 0.66 and 0.934 ^(^ 23 ^,^ 27 ^)^. These results reinforce the association between glycemic control and HbA1c, providing important evidence for monitoring DM. However, it is necessary to discuss the divergences found between these studies and the existing literature. 

The International Consensus on the Use of CBGM ^(^ 3 ^)^ establishes the need for at least 14 uninterrupted days of data with approximately 70% of CBGM readings during this interval for the purpose of time-on-target analyses. In this context, two studies in this review presented data intervals of less than 14 days ^(^ 25 ^,^ 33 ^)^, which could possibly reflect on the quality of their results. 

It should be noted that there is still no consensus in the literature on the use of time on target with glycemia values from SMCBG and, therefore, there is no consensus on the period of data necessary for research using metrics arising from self-monitoring.

The present review found only one study that used SMCBG data to calculate time on target, time above target and time below target correlating with HbA1C. In fact, this study adopted a new terminology, the target point, since the SMCBG values reflect measurements determined by the person living with diabetes at a given point in time ^(^ 26 ^)^. 

There was also divergence between the different target times examined in the chosen studies. Although all of them presented the time on target (70-180 mg/dl), the demand for investigation on other different times is identified in the literature, as this metric, by itself (time on target 70-180 mg/dl), is not an adequate description of overall glycemic control. It is also pertinent to quantify the times below and above the target, using some severity thresholds for each level ^(^ 3 ^)^. 

Therefore, it is necessary to even calculate the percentage of time spent below target level 2 (<54 mg/dl) with urgency for action; time below target level 1 (<70 mg/dl); optimal time on target (70-140 mg/dl); time above the level 1 target (>180 mg/dl) and time above the level 2 target (>250 mg/dl) with urgency for action ^(^ 3 ^)^. In this context, six studies ^(^ 22 ^,^ 31 ^-^ 34 ^,^ 36 ^)^ corroborated what is determined in the literature. 

Most of the studies included in this systematic review ^(^ 22 ^-^ 24 ^,^ 26 ^-^ 28 ^,^ 31 ^-^ 34 ^,^ 36 ^)^ also investigated, through the correlation between time on target and HbA1c, the impact of a certain proportion on time spent in the target range in HbA1C. 

A study found that a time on target of 70% corresponds on average to an HbA1C of 7% and the lower the proportion of time on target, the higher the HbA1c value will be, with a time on target of 50% equivalent to an HbA1c of 8% and a time on target of 30% at an HbA1c of 8.7% ^(^ 22 ^)^. 

A study carried out in a pediatric population ^(^ 24 ^)^ found that an ideal time on target (70-140 mg/dl) at 50% corresponds to an HbA1c of 6.5%; another study, carried out with children and adolescents ≤18 years old, found a similar result, a time to target of 70-180 mg/dl of 55.1% for an HbA1c of 7% ^(^ 27 ^)^. Another study found a higher proportion of time at the 70-180 target, of 65% for an HbA1c of 7% ^(^ 32 ^)^. 

It is noteworthy that a study carried out in a population aged between 20 and 69 years old found a 0.5% decrease in HbA1c from 7.5% to 7%, when there was an improvement in the proportion of time on target (70-180 mg/dl) from 52.9% to 58.8% ^(^ 28 ^)^. 

The only research carried out with pregnant women living with DM1 showed that to reach HbA1c of 6%, 6.5% and 7%, an average time on target (60-140 mg/dl) of 78%, 74% is required. and 69%, respectively ^(^ 30 ^)^. And another study carried out with patients with DM1 and DM2 undergoing insulin treatment found that of the 530 participants, 26% (n=139) had a target time (70-180 mg/dl) in 70% and of these 139 participants, 79.8% (n=111) had an HbA1c of 7% ^(^ 23 ^)^. 

Only one study differentiated the impact of time on target on HbA1c in patients with DM1 from the population with DM2, finding that a time on target of 70% corresponds to an average HbA1c of 6.9% in people with DM1 and in the same proportion (70%) corresponds to an average HbA1c of 7.1% for people with DM2 undergoing treatment with oral hypoglycemic agents or insulin ^(^ 34 ^)^. 

The divergences evidenced between these studies ^(^ 22 ^-^ 24 ^,^ 26 ^-^ 28 ^,^ 31 ^-^ 34 ^,^ 36 ^)^ in relation to the different proportions for a given time on target that corresponds to an HbA1c ≤7% are possibly the result of ethnic and pathophysiological differences. of each participant, since HbA1c has limitations in relation to age, erythrocyte lifespan and can be affected by factors other than hyperglycemia, such as in some diseases such as anemia and chronic kidney disease ^(^ 38 ^)^. 

Therefore, the study that showed a higher proportion of time on target (80%) for an average HbA1c of 7% was carried out in an older population between 30 and 80 years old, which probably had greater pathophysiological risks among participants ^(^ 36 ^)^. 

There is a need for future studies that evaluate HbA1c goals according to the age group of the participants and their comorbidities, as is already established in some guidelines for the care and treatment of DM ^(^ 39 ^)^. 

It is noteworthy that the objective of this systematic review was not to seek evidence of the possibility of replacing HbA1c with time on target, on the contrary, it was to track in the literature whether there is a correlation between this metric and HbA1c, seeking to better understand how the relationship between these two tools in the glycemic control of people living with DM.

It should be noted, in this context, that the results of this review show that the correlation between time on target and HbA1c indicates the relevance of still using HbA1c as a measure to assess the risk of complications related to diabetes, however, together with time on target, with the aim of enhancing the identification of risks for micro and macrovascular complications of DM.

A limitation is the identification of only one study with glycemia data from the SMCBG ^(^ 26 ^)^ and, despite this finding a correlation between target points and HbA1c, it is too early to ratify this metric to assess glycemic control in patients who make use of SMCBG devices, unlike those that use CBGM. 

Therefore, the need for more studies that analyze time on target and other metrics with SMCBG glycemia data and its correlation with HbA1c emerges, essentially because CBGM is a technology accessed in a restricted way by a small part of the population. people living with diabetes, and the SMCBG is therefore still widely used.

Another limitation was the unfeasibility of carrying out a quantitative (statistical) synthesis of the results due to the significant heterogeneity of the methodological configurations between the selected studies, mainly in relation to statistical analysis to evaluate the correlation between time on target and HbA1c.

As an impact factor in clinical practice, time on target and its other metrics can be used by healthcare professionals as a tool to assess patients’ glycemic control in the short, medium and long term, differently from and in addition to HbA1c. Furthermore, it is a tool that can be used as a way to educate and empower patients to identify states of hypoglycemia and hyperglycemia , especially when at levels <54 mg/dl and >250 mg/dl, and also to manage more effectively your own glycemic control, since the greater the proportion of time on target (70-180 mg/dl) or (70-140 mg/dl), the closer the HbA1c values will be between ≤7% ^(^ 22 ^-^ 24 ^,^ 26 ^-^ 28 ^,^ 31 ^-^ 34 ^,^ 36 ^)^. 

In this context, the finding of a correlation between time on target and HbA1c in the present review may provide implications for the advancement of scientific knowledge in the health area, such as the use of this new metric as a complementary measure to HbA1c in the assessment of glycemic control, enabling development of more effective therapeutic strategies. Furthermore, the present investigation may encourage the conduct of additional studies with the aim of deepening the understanding of this correlation.

## Conclusion

It is concluded that there is a statistically significant correlation between time on target and time above target with HbA1c. The greater the proportion of time in the appropriate glycemic range, the closer to or below 7% the HbA1c will be. Furthermore, its correlation with HbA1c suggests a potential impact on clinical practice, allowing the development of more effective therapeutic strategies by health professionals and managers. This discovery also encourages the development of future research to obtain a more comprehensive understanding of this correlation and its clinical implications.
